# Poverty is associated with both risk avoidance and risk taking: empirical evidence for the desperation threshold model from the UK and France

**DOI:** 10.1098/rspb.2024.2071

**Published:** 2025-02-05

**Authors:** Benoît de Courson, Willem E. Frankenhuis, Daniel Nettle

**Affiliations:** ^1^ Max Planck Institute for the Study of Crime, Security and Law, Freiburg im Breisgau, Germany; ^2^ Leiden University, Leiden, The Netherlands; ^3^ Département d’études cognitives, Institut Jean Nicod, Ecole Normale Supérieure, Université PSL, EHESS, CNRS, Paris, France; ^4^ Institute for Biodiversity and Ecosystem Dynamics, University of Amsterdam, Amsterdam, The Netherlands; ^5^ Department of Social Work, Education and Community Wellbeing, Northumbria University, Newcastle upon Tyne, UK

**Keywords:** poverty, optimal foraging, risk taking, modelling

## Abstract

In situations of poverty, do people take more or less risk? One hypothesis states that poverty makes people avoid risk, because they cannot buffer against losses, while another states that poverty makes people take risks, because they have little to lose. Each hypothesis has some previous empirical support. Here, we test the ‘desperation threshold’ model, which integrates both hypotheses. We assume that people attempt to stay above a critical level of resources, representing their ‘basic needs’. Just above this threshold, people have much to lose and should avoid risk. Below, they have little to lose and should take risks. We conducted preregistered tests of the model using survey data from 472 adults in France and the UK. The predictor variables were subjective and objective measures of current resources. The outcome measure, risk taking, was measured using a series of hypothetical gambles. Risk taking followed a V-shape against subjective resources, first decreasing and then increasing again as resources reduced. This pattern was not observed for the objective resource measure. We also found that risk taking was more variable among people with fewer resources. Our findings synthesize the split literature on poverty and risk taking, with implications for policy and interventions.

## 1. Introduction

In situations of poverty, do individuals tend to take more or fewer risks? On this question, there are, as Banerjee puts it, ‘at least two distinct and, prima facie, inconsistent views’ [1]. The first is that poverty makes individuals ‘vulnerable’: they have barely enough to make ends meet and would suffer too much from a resource loss. Therefore, they avoid risk. The second is that poverty makes individuals ‘desperate’: they have little to lose and are ready to gamble to have a chance to get out of poverty, since their situation cannot get much worse. Therefore, they take more risks. Even though these two views predict opposite associations between levels of resources and risk taking, both can be found in theories across the social sciences (for examples of the view that poverty increases risk taking, see [2–6]; for examples of the view that poverty decreases risk taking, see [7–10]). Both views have also been used to make sense of empirical findings. The idea that people in poverty avoid risk has been invoked to explain the lack of professional specialization [10], a reluctance to adopt new technologies or to invest in education [7], and even the persistence of poverty [7,11]. On the other hand, the idea that people in poverty have ‘little to lose’, and therefore seek risk, has been invoked [3,5,12] to explain higher prevalence of crime [13] and gambling [14] in deprived populations.

The empirical record is also mixed [15–18]. In high-income countries, most cross-sectional studies have found that individuals with a lower income or wealth take fewer risks in experimental gambling tasks (e.g. [19–21] a review, see [22]). In low-income countries, some studies have also reported less risk taking [11], but others found no association [23–25], or even more risk taking. For instance, the poorest Indian farmers were found to be extremely willing to take risks [26]. Among poor Madagascar farmers, food insecurity was the best positive predictor of risk taking in hypothetical gambles [27]. Another study used the choice between drought-resistant camels and more productive but riskier small livestock, as a proxy of risk taking among four herder groups [28]. In three of the four groups, the poorest households kept mostly riskier small livestock. To sum up, there is a crucial inconsistency: two bodies of work propose and document exactly opposite associations between poverty and risk taking. Both views are intuitively appealing, and both have support in the empirical record. Both are relevant to explaining key social phenomena, such as occupational choices and crime.

Optimal foraging theory, and risk-sensitive foraging in particular, can resolve this conundrum [29,30]. In a scenario first modelled by Stephens [31], a ‘small bird in winter’ aims to acquire enough calories to get through the night. Just above the ‘starvation threshold’—where the bird is likely to survive, but only just—it should avoid risks, so as to not fall below it. However, below this threshold, it should take risks to have a chance to keep its head above water. Thus, low compared to high energetic resources will be associated with either greater risk avoidance or greater risk taking, depending on how low they are. Analogous applications of this idea to humans in situations of resource scarcity have emerged independently in disparate fields of research, including psychology [32,33], agricultural economics [34,35], development economics [36], anthropology [37] and political science [38]. In our papers, we [29,30] applied this same logic to criminal behaviour in situations of poverty. We assumed that individuals have a ‘desperation threshold’ representing ‘basic needs’ that they try to always meet. We elaborate on the desperation threshold model and its predictions in §2.

The desperation threshold model has been tested in lab experiments [33,39–44]. Participants—students or online participants from North America or the United Kingdom—typically play a game that includes an artificial threshold, such as a minimum number of points needed to obtain a monetary payoff at the end of the game. Participants tend to behave in accordance with the theoretical prediction, taking fewer risks when their resource level is above the threshold, and more below. These findings suggest that people are able to adjust their behaviour when confronted with a threshold. But they tell us little about behaviour in natural environments. Do such thresholds exist outside the lab? Do they affect the behaviour of a sizeable fraction of the population?

Evidence for the predictions of the desperation threshold in real-world settings is scarce, in part because cross-sectional studies are often ill-suited to testing threshold effects. Such studies tend to model risk taking as a linear function of resources, whereas the desperation threshold predicts a nonlinear mapping (a U- or V-shape): poverty should reduce risk taking up to some point, and then increase it. Nevertheless, several studies are informative. For instance [45], estimated risk taking by quintiles of income and wealth in the Health and Retirement Study, a representative panel of Americans over age 50. Consistent with the desperation threshold, people in the poorest and the richest quintiles, whether measured in income or in wealth, were ready to take the most risks. Recently, Akesaka *et al*. [46] documented in the same dataset that those who strongly depended on social security—those with fewer resources—were ready to take significantly more risks the day before receiving welfare checks, when they are most likely to be below the threshold than at other times.

In anthropology, Kuznar [47] presented evidence of a U-shape between herd value and risk taking—but the small size of the sample (23 Andean farmers) limits statistical inference. Caballero [48] estimated a subsistence threshold in extremely deprived neighbourhoods of Bogota and found preliminary evidence of a jump in risk taking at that point. But again, the sample size was not sufficient to draw firm conclusions. In principle, though, any dataset that includes measures of resources and risk taking could be used to test the hypothesis, as long as there are enough people above and below the desperation threshold. In sum, there is some evidence from diverse populations of U- or V-shaped relationships between material resources and risk tolerance, but the number of studies is limited and many of them are based on small samples.

In this article, we first offer a succinct formalization of the desperation threshold model, from which we derive the predicted nonlinear relation between resources and risk taking. Then we test those predictions using the Changing Cost of Living dataset [49], a survey of British and French adults that includes questions about participants’ levels of resources across time, as well as a measure of risk taking. Moreover, these questions concerned not only income but also unavoidable costs and subjective feelings of poverty. Thus, we can test the prediction using an objective measure of resources, and a subjective one, the feeling of resource adequacy.

## 2. Theory

The desperation threshold idea can be summarized as follows: humans have a strong preference for having at least some amount of resources that represent their ‘basic needs’. Above this level, they continue to derive utility from resources, but this is less important than keeping their basic needs secured. We can formalize this threshold with a utility function. The initial set of models captured this idea with a jump in the utility function [50], or even a step function, representing life and death [31]. Here, we assume a more general sigmoid shape. The utility function features a steep region, representing that at some point resources are particularly valuable because they secure basic needs. Below the threshold, the utility function is relatively flat, representing the intuition that one has ‘little more to lose’ once basic needs are not reached. Above the threshold, we assume that utility increases linearly with resources.

**Figure 1.**
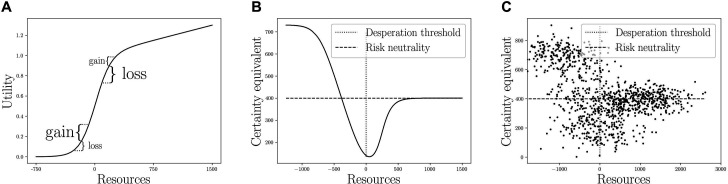
Summary of the model and predictions. Panel (A) is the utility function we assume to represent the desperation threshold. Panel (B) is the resulting certainty equivalent of the risky decision—the minimum guaranteed amount of money one would accept instead of taking a 50% chance to win €800—depending on resources. Certainty equivalent being a measure of risk taking, this is the association we predict between risk taking and resources. Panel (C) represents the same as panel (B) when resources are observed with a large noise (s.d. = €500) and certainty equivalents with a smaller noise (s.d. = €50). This is the basis of our second prediction, that risk taking should vary more among individuals with low resources.

Our utility function is therefore


$$U(x)=\frac{1}{1+e^{-x}}+\frac{x}{50}1_{x\;>\;0},$$

where x represents resources and the threshold is placed at 0 $1_{x>0}$ being an indicator function, whose value is 1 when x>0 and 0 otherwise. Figure 1A represents this utility function and highlights the central result of the model. Below the threshold, the function is convex: one has more to win than to lose and should therefore take risks. Above the threshold, the function is concave: one has more to lose than to win and should therefore avoid risks. It should be noted that the desperation threshold does not predict a change in ‘risk preference’ strictly speaking from an economic point of view: the taste for risky outcomes is unchanged. Rather, Lybbert [51] has coined the term of ‘risk response’ to threshold effects, which applies here. Thus, we use the terms ‘risk taking’ and ‘risk avoidance’, rather than ‘risk proneness’ and ‘risk aversion’.

In figure 1B, we plot the ‘certainty equivalent’ depending on resources. Concretely, we assume that an individual has the utility function shown in figure 1A, and we let her decide between €x for sure and a 50% chance of getting €800. We vary x from 0 to 800 and we plot in figure 1B the minimum value of x she would take. This represents the value she attributes to the risky choice and can therefore be used as a measure of risk taking. If the certainty equivalent is more than 400, the person is taking risk; if it is less than 400, they are avoiding risk, and if it is exactly 400, they are risk neutral. In the task used in the Changing Cost of Living survey (see next section), the certainty equivalent represents the point where participants would switch to the safe option. The resulting prediction (figure 1B) is that below the threshold, people should take risks even when the expected value of the certain option is higher than that of the risky options, whereas just above the threshold they should avoid risks even when the certain payoff has a worse expected value than the risky option. Note that the switch to risk taking occurs below the threshold here, since participants can only gain resources in the task. The switch from risk avoidance to risk taking is reached around x=−400, where €400 with certainty means ending up precisely at the desperation threshold. Thus, our first prediction is that risk taking should be a V-shape function of resources (figure 1B).

Now, what if resources are only imperfectly observed? As risk taking varies abruptly with resources around the desperation threshold, it is crucial to tell apart individuals just above the threshold from those just below. In practice, this might not be realistic: resource levels are not perfectly measured, and the threshold may vary from individual to individual. In figure 1C, we present our prediction if resources are observed with a high noise (s.d. = €500) and certainty equivalents with a low noise (s.d. = €50). The V-shape is not visible to the naked eye anymore, but we obtain a triangle-shaped scatter plot. This is the basis of our second prediction: risk taking should be more variable in the lower part of the resource distribution than the higher part. This is because the lower part comprises some individuals just above and some individuals just below the threshold, with opposite levels of risk taking.

To sum up, the desperation threshold model makes two predictions. First, that risk taking should follow a V-shape of resource levels, with both the poorest and the richest participants taking more risks than average (P1). Second, that risk taking should vary more between individuals at low resource levels (P2), since one should find both ‘vulnerable’ participants avoiding risks, and ‘desperate’ ones taking risks. In §3c, we explain how we tested P1 and P2 using the Changing Cost of Living dataset.

## 3. Methods

### (a) Panel

We used the data collected for the Changing Cost of Living study (for a complete description of this data collection, see [49]; protocols available at https://osf.io/e8g3p). In September 2022 the authors recruited a panel of 232 French and 240 British adults over the age of 25. Participants were invited to complete a survey once a month for 12 months. On average, participants completed 10.05 of the 12 surveys each (s.d. 2.98). In August 2023, when the study ended, 157 (67.7%) and 216 (90%) of the original participants responded. Electronic supplementary material, table S1 shows participant demographics. The panels were not nationally representative and were skewed towards the low end of their respective national income distributions, especially in France (see [49] for details).

### (b) Measures

The full set of measures is described in the preregistered initial (https://osf.io/x26mf) and supplementary (https://osf.io/rj683) protocols of the study. The data were collected for multiple studies (in particular [49]), and we only use some of its measures.

#### (i) Objective resources

Participants reported the amount of income received into their household in the reference month (i.e. net of taxes and including benefits). UK figures were converted to euros at a purchasing-power-parity rate. The mean income of participants was 3437€ and the median 3000€ (s.d. = 2117.1). For costs, participants reported the amounts paid out for rent/mortgage, water, residence-based taxes and energy (electricity, gas and oil) in the previous month. We summed these amounts to obtain an estimate of unavoidable living costs. We logged income and cost variables with a base 2 (adding €1 because of zeroes), to reduce positive skew and represent the fact that resources have diminishing returns. Our objective resources variable is the difference between the log-transformed income and log-transformed unavoidable costs. Since the difference in logs is the log of the ratio, this variable measures the proportional relationship of household income to unavoidable costs. Thus, a value of zero means that income just covered unavoidable costs; a value of 1 that income was twice unavoidable costs; and a value of 2 means that income was four times unavoidable costs. Negative values (1.6% of cases) indicate failure of income to even cover unavoidable costs.

#### (ii) Subjective resources

Participants were asked three questions about their subjective risk of losing resources: their subjective risk of destitution, their subjective risk of losing ‘a suitable place to live’ and their subjective risk of losing ‘a suitable employment’. Participants answered these three questions on a 0−100 scale, which we summed and reverse coded to compute our subjective resources measure. The three variables had a Cronbach’s alpha of 0.87. To avoid right-skew (a large number of participants reported almost zero on those three measures), we applied a square root transformation. Subjective resources were moderately correlated with objective resources (r= 0.27, *p* < 0.001).

#### (iii) Risk taking

Participants were asked whether they preferred a 50% chance of getting €800, or €x for sure, with x being increased by €100 from €100 to €700. We used the number of risky bets (choosing 50% chance of getting €800) that participants preferred as our measure of risk taking. If participants were perfectly consistent, this measure would be proportional to the minimum certainty equivalent that we presented in figure 1B. But it is more robust to a ‘trembling hand’ of the participants: if a participant mistakenly refuses the least risky bet but is actually risk neutral, then our measure will almost be correct (3 instead of 4), while the minimum certainty equivalent would have yielded 1.

On average, participants accepted 2.31 of the 7 bets (s.d. = 1.6). Participants were weakly-to-moderately stable over time in their risk taking: the intra-class correlation coefficient (ICC) was 0.48.

#### (iv) Time-discounting

Participants were asked whether they preferred €100 now or €x 90 days from now, with *x* ranging from 110 to 170. We used the number of immediate choices as our time discounting measure. We use this variable in our exploratory analysis (see below), for contrast with the results we obtained with risk taking.

### (c) Analysis strategy

We first investigated descriptively the relationship between resources and risk taking. We then ran five confirmatory tests of our predictions relating risk taking to resource levels. These analyses were preregistered here: https://osf.io/g4x8t/. In §4, we present each test twice, using, respectively, objective and subjective resources. We corrected *p*-values using the Holm–Bonferroni method to control the error rate. These tests are divided into two distinct groups, differing in their level of severity to test our hypothesis (see below). The two groups relate, respectively, to P1 and P2 (see §2).

In our first group of analyses (analysis 1), we predicted that risk taking would follow a V-shape against resources. First, we fitted mixed-effects polynomial models, to test for evidence of a nonlinear relationship between resources and risk taking. Second, we fitted segmented linear models, to estimate the association below and after a ‘changepoint’, fitted with maximum likelihood. This approach is less standard in psychology and has been judged problematic in exploratory analyses [52]. However, our analysis is confirmatory, and our model prediction is closer to a broken-stick relationship (figure 1B) than a smooth polynomial. We constrained the model to have the two regression lines connected, by fitting the following formula: $risk\_taking=\beta_{0}+\beta_{1}(r-cp)(r\leq cp)+\beta_{2}(r-cp)(r>cp)+\text{controls}$, where cp is the changepoint and r stands for the resources. The polynomial and the segmented models are two different ways to represent the predicted V-shape. We see them as two different tests of the same prediction.

In these analyses, we included random effects of participants and fixed effects of age and gender, two variables known to influence risk taking [5,20].

Our second group of analyses (analysis 2) tested the less specific prediction that risk taking should be more variable in individuals with fewer resources. Our reasoning here was as follows: our resource measures may be too noisy for discriminating when individuals are just below the threshold and when they are just above, especially since the threshold might vary between individuals. In this case, we might not be able to identify a single switch point between risk avoidance and risk taking, but we should still expect a mixture of risk takers and risk avoiders at the bottom of the resource distribution, whereas risk preference should be more homogenous higher in the distribution (figure 1C). We therefore tested in three ways whether variance in risk taking was higher among individuals with fewer resources. Specifically, we tested:

- whether variance in risk taking was higher among individuals reporting that ‘managing financially is very difficult’;
- whether squared residuals of a linear model were higher at the bottom of the resource distribution; and
- whether participants with lower resources were less stable over time in their risk taking.

This second group of analyses represents a less severe test of the model than the third one, in the sense that the predicted result could be obtained under less stringent conditions, and, as a result, more alternative explanations could be proposed (see §5).

Finally, we ran an exploratory analysis that was not preregistered. There, we used all the available resource variables to isolate the most deprived individuals, according to different criteria. We computed descriptive statistics of risk taking in these categories: the mean, variance and frequency of extreme values, and compared them to the full sample population. We also contrasted the results with the ones obtained with richest individuals and used time discounting instead of risk taking.

## 4. Results

### (a) Descriptive analysis

To visualize how risk taking and resources were related, we plotted the average level of risk taking depending on the answer to the question ‘How are you managing financially?’. We obtained (electronic supplementary material, figure S1) an approximately linear, increasing trend: the easier to manage, the more risks, on average. This could however hide the fact that, as we suggested, those who report that managing financially is ‘very difficult’ include a minority of risk takers, hidden behind a majority of risk avoiders.

**Figure 2.**
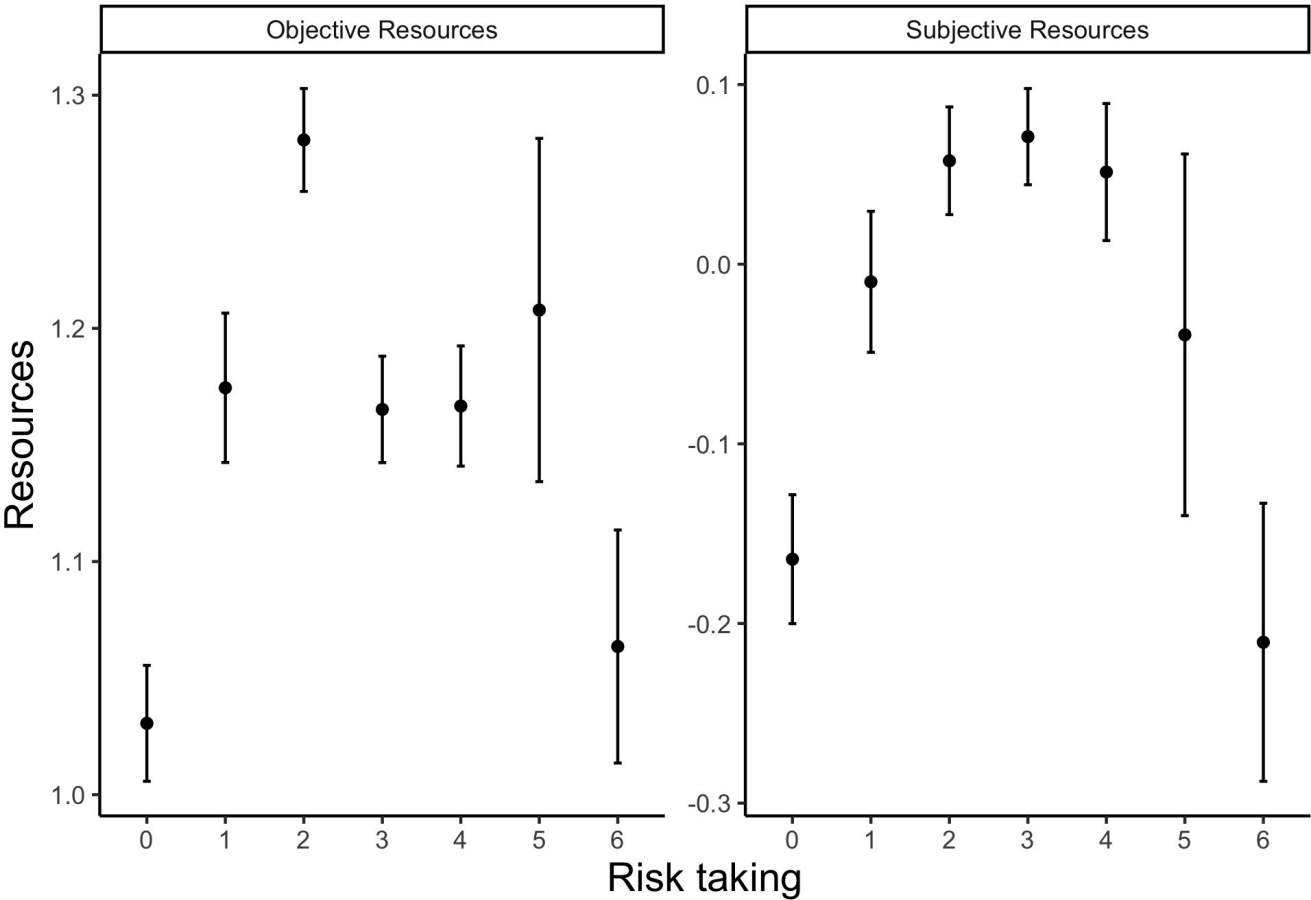
Objective and subjective resources are summarized by risk taking answer. In these plots, we have pooled together the participants who accepted six and seven risky bets, to have a large enough group. The error bars represent 1 s.e. of the mean.

To investigate this possibility, we examined the average values on our two resource measures of people choosing each of the possible numbers of risky options (0−7). Figure 2 shows the results. An inverted V-shape is clear in both cases: both the participants who were ready to take *the least* and *the most* risks had on average fewer objective and subjective resources.

Finally, we looked at the prevalence of extreme risk taking among the 5% with the lowest levels of objective and subjective resources (table 1). We defined participants as ‘risk avoiders’ when they accepted no bets, and as ‘risk takers’ when they accepted more than four bets. We use this term because a participant accepting more than four bets necessarily preferred a risky bet to a safe one that had a higher expected payoff (for instance, a 50% chance of getting 800€, rather than 500€ for sure). In electronic supplementary material, Table S5, we expand this table, adding more descriptive statistics of risk taking.

**Table 1.** Extreme risk taking prevalence among participants low on resources. Asterisks denote the *p*-values of tests comparing the category with the rest of the sample, using chi-squared tests. Asterisks represent significance levels: *p < 0.05; **p < 0.01; ***p < 0.001.

categories	% of risk takers	% of risk avoiders	*n*
full sample	6	17.4	4882
bottom 5% in objective resources	8.4	34.7 ***	242
bottom 5% in subjective resources	12 ***	23.7 *	243

Risk avoiders were more common in the bottom 5% than in the full sample. This was true whether resources were defined objectively or subjectively. Risk takers were also more common in the bottom 5% than the full sample. Again, this was true whether objective or subjective resources were used, but it was particularly marked for subjective resources. The difference in prevalence of risk takers was only significant with subjective resources, but since risk takers were about three times rarer than risk avoiders, the power of these tests was much lower. Also, risk taking was on average lower (significantly for objective resources), but the variance in risk taking was, respectively, 37 and 44% higher than in the full sample (*p* < 0.001 in both cases) (see electronic supplementary material, table S5).

### (b) Analysis 1

#### (i) Polynomial regressions

We fitted a cubic polynomial of resources on risk taking, with a random effect for participants and fixed effects for age and gender. Here and in all the following regression models, we found the usual association for age and gender, with women and older individuals taking fewer risks (for the linear model, we obtained β= −0.19, p= 0.005 for women, β= −0.085, p= 0.01 for standardized age).

**Figure 3.**
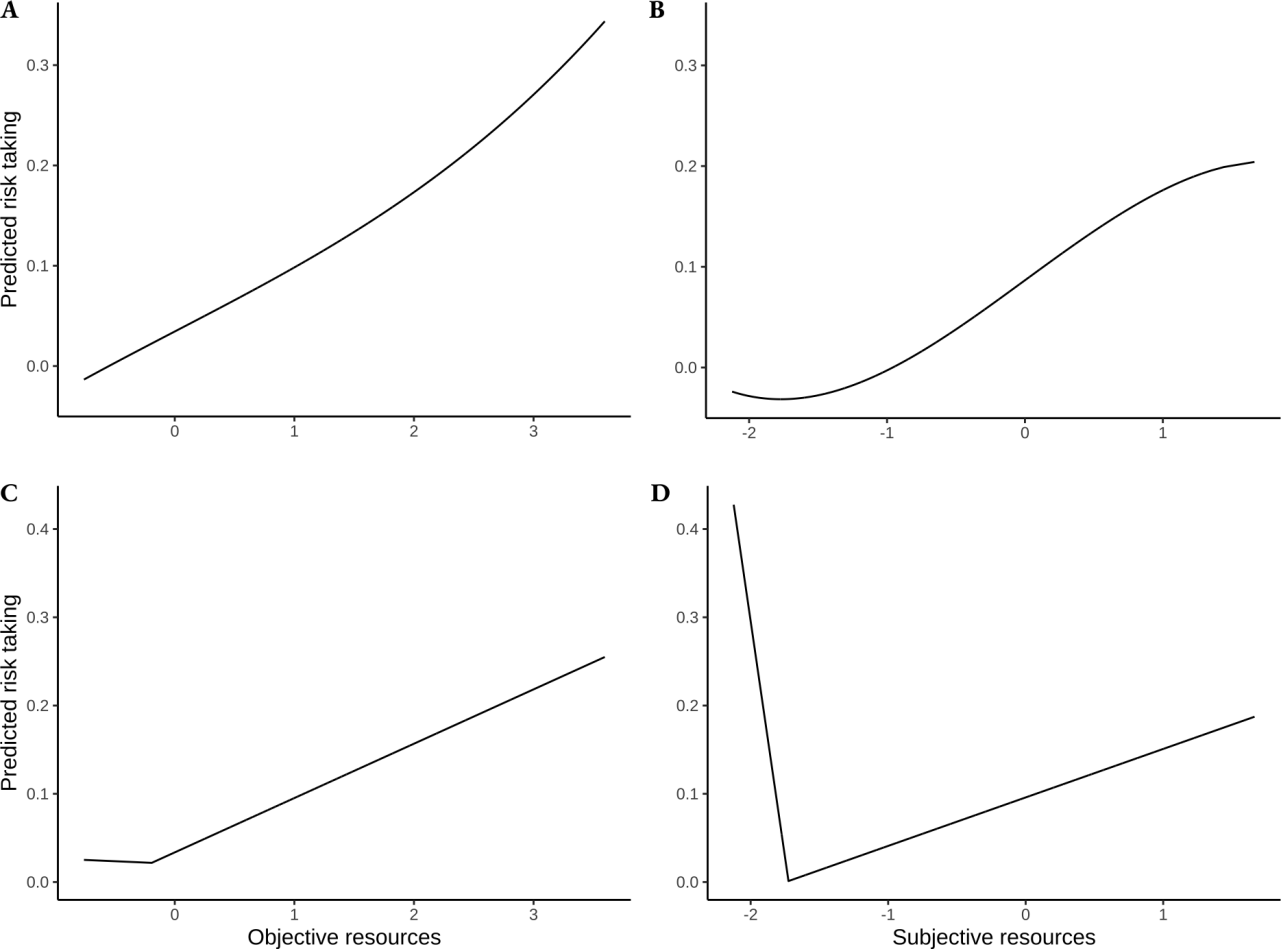
Risk taking predictions by the nonlinear statistical models, with (A,B) polynomial and (C,D) segmented regressions, for (A,C) objective and (B,D) subjective resources.

We predicted that the fitted polynomial would have an inflection point in the lower half of the resource distribution. This prediction was supported with subjective resources, but not with objective resources, which showed an almost linear relationship (figure 3A,B). We predicted that a quadratic or cubic model would fit the association of resources to risk taking better than a linear one. For objective resources, this was not the case: both the quadratic and the cubic model had a higher Akaike information criterion (11 587.7 and 11 588.5, respectively) than the linear one (11 585.7). Neither can reject the linear model in a likelihood ratio test ($\chi^{2}$ = 0.032, p= 0.859 for the quadratic model, $\chi^{2}$= 0.032, p= 0.537 for the cubic one). As a preregistered follow up analysis, we fitted higher-degree polynomials, looking for the model with the least AIC. No model had a lower AIC than the linear one.

With subjective resources, a cubic model had a lower AIC (11 603.1) than the linear one (11 603.4), the quadratic one (11 604.3) and any higher degree model. However, the superior fit of the cubic model over the linear one was not significant in a likelihood ratio test (4.31, p= 0.232).

#### (ii) Segmented mixed models

We fitted segmented mixed models between resource variables and risk taking. The changepoint was fitted by maximum likelihood, testing all possible values to identify the breakpoint giving the smallest deviance, which is a smaller-is-better measure of model fit. In electronic supplementary material, figure S2, we plot the deviance of the model, depending on the changepoint location.

Tables S2 and S3 show the scaled coefficients and the associated significance two-sided *t*-tests, for objective and subjective resources, respectively. Figure 3C and 3D show the patterns between resources and risk taking predicted by the fitted models. With objective resources, we obtained the predicted V-shape (see figure 3C). The slope of the association was significantly different from zero above the changepoint, but not below. The changepoint was found at the extreme bottom of the distribution (99% of the observations are above).

With subjective resources, all our predictions were supported. We obtained a V-shape, with resources having a significantly negative effect below and significantly positive above the threshold. After correction for multiple comparisons with objective resources, both tests remained significant (p= 0.029 and p= 0.034). As predicted, the changepoint was found at the lower end of the resource distribution (3.9% of the data points are below it). The effect below the threshold was 19 times stronger than the effect above the threshold. We had not predicted a stronger effect below the changepoint in our preregistration, but this is clearly an implication of the desperation threshold model (figure 1B). Electronic supplementary material, figure S2 revealed that one could account almost as well for the data with a slightly higher changepoint (11% of the data points were below it). As a robustness check, we checked that our predictions were also supported with this alternative changepoint. Electronic supplementary material, table S4 presents the scaled coefficients of this model. A V-shape was also found, with a significant effect on both sides of the changepoint.

### (c) Analysis 2: do individuals with low resources vary more in risk taking?

#### (i) Is there more variance in risk taking among close-to-the-edge participants?

In our second analysis, we predicted that there would be more variance in risk taking at the bottom of the resource distribution. We tested this prediction using the financial strain question, the objective and the subjective resources variables. As predicted, individuals who report that managing financially is ‘very difficult’ had a 35% higher variance in their risk taking answers (*F*(4590,250) = 0.74, p< 0.001). This also applied, to a lesser extent, for people who reported that it was ‘quite difficult’ to manage financially (electronic supplementary material, figure S1B).

To test the same question with our (continuous) resource variables, we fitted linear regressions between resources and risk taking, keeping age and gender as controls, but without a changepoint and without random effects, so as not to neutralize the between-individual variance. Then, we predicted that squared residuals would decrease with resources in a new linear regression, that is, that the absolute deviation from the line of best fit would be larger at the bottom of the resource distribution. Since this analysis tests for the same prediction as the one above using the financial strain question, we apply a Holm–Bonferroni correction to the three *p*-values. The prediction was clearly met for both objective and subjective resources (β=−0.09 and β= −0.06, respectively, p < 0.001 and p < 0.001).

**Figure 4.**
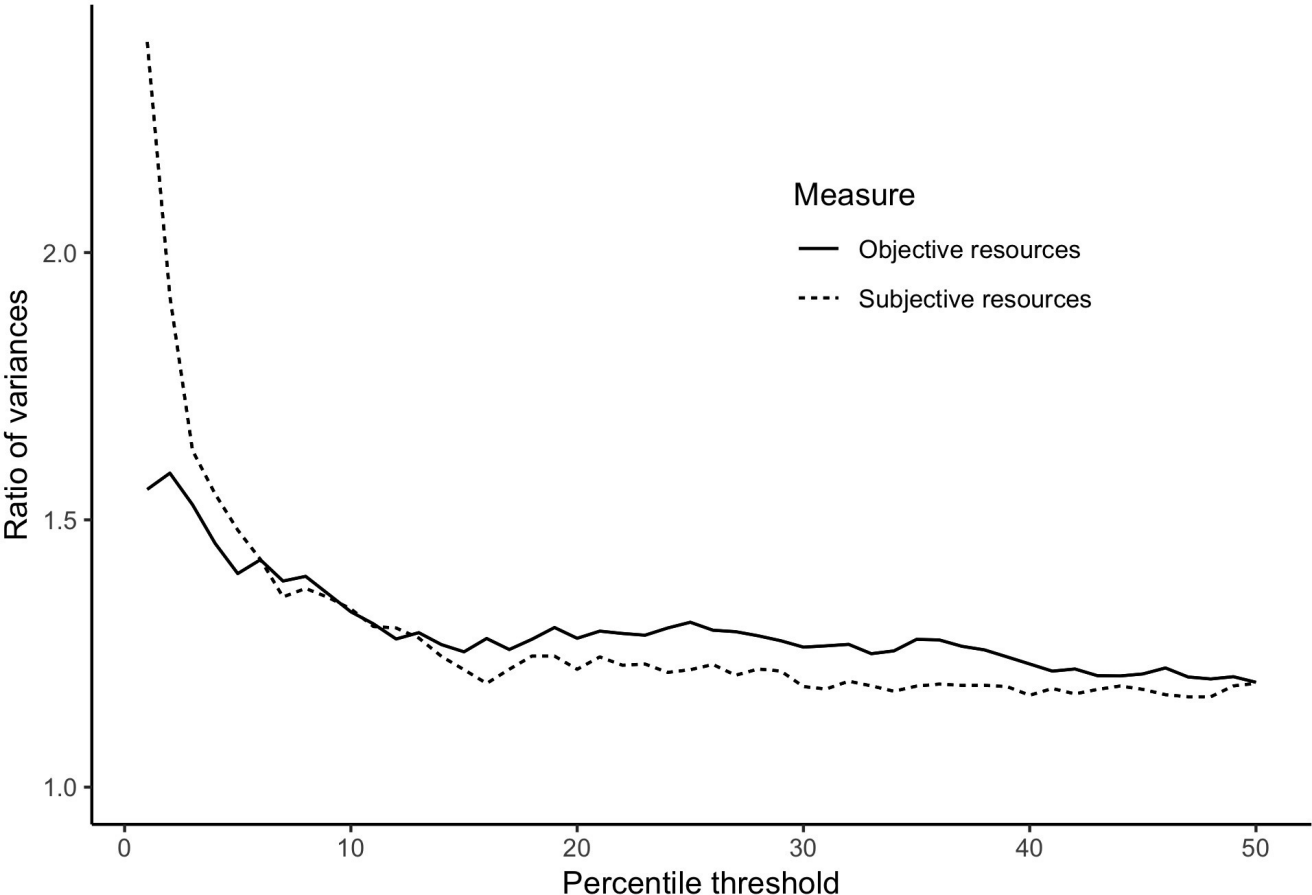
Ratio of variances in risk taking below and above resource thresholds set at different levels, from the first percentile to the median. For any threshold, the difference in variance is significant (*p* < 0.05).

We visualized this effect by comparing variance in risk taking below and above some resource threshold, varying this threshold from the first percentile of the resource distribution to the median value (figure 4). For any threshold below the median, the variance was at least 17% higher in the bottom part of the distribution. This variance soars as the threshold goes towards zero, in particular using subjective resources.

#### (ii) Are participants with low resources less stable over time in their risk taking?

Finally, we tested a slightly different prediction: participants with fewer resources should sometimes hover around the threshold and should then alternate between taking and avoiding risks. We would thus expect that an individual with fewer resources will vary more in risk taking over time. We computed the intra-personal variance in risk taking over all time periods for every individual and fitted a linear model between this variance over time and the average resource value.

For objective and subjective resources, the association was in the predicted direction. It was significant with objective resources (standardized β= −0.14, p= 0.004), but not with subjective resources (standardized β= −0.058, p= 0.22). We must note that the statistical power of these two tests was much lower than the previous ones: since they aggregated all the responses from the same individual, they are based on only 485 data points, against 4817 before.

### (d) Ruling out alternative explanations

We were interested in knowing whether our finding was specific to participants with fewer resources and to risk taking. Therefore, we replicated table 1 on the top 5% answers in terms of objective and subjective resources (electronic supplementary material, table S5, lines 2 and 3), and using the time discounting variable of the dataset, instead of risk taking (electronic supplementary material, table S6). We preregistered this analysis as a follow-up (https://osf.io/54hfq).

With time discounting, we predicted (i) that there would be more individuals with high time discounting (defined as choosing only immediate rewards), but (ii) not more individuals with low time discounting (defined as never choosing immediate rewards) in the deprived categories than in the full sample and (iii) that similarly, variance would not be more than 30% higher (30% being the lowest difference we found in risk taking). In both categories, high-time discounting was more than twice as frequent in the deprived categories. In the bottom 5% of objective resources, our two other predictions were not supported: variance in time discounting was 44% higher than in the full sample, and low time discounting was slightly more frequent (22.4%) than in the full sample (18%). With subjective resources, all predictions were supported: variance was 27% higher than in the full sample, and low-time discounting was less frequent (15%) than in the full sample (18%). Thus, it seems that our observation that low resources were associated with extreme risk taking was specific to risk taking and did not reveal a tendency to make extreme decisions in other domains.

Finally, as another test of comprehension, we examined whether individuals with fewer resources were more likely to produce inconsistent answers in the risk questions. Among the full sample, we categorized 6.5% of the answers as inconsistent, in the sense that the participant refused a bet that was more profitable than another bet they accepted. However, neither objective nor subjective resources were correlated with consistency (r= 0 and r= 0.05, respectively), providing no evidence for differences in comprehension.

## 5. Discussion

### (a) Summary of results

In a panel of adults from France and the UK, we investigated the association between (lack of) resources and risk taking. We found clear evidence that having low resources is associated with a higher variance in risk taking (figure 4), and with a large increase in both extreme risk avoidance and extreme risk taking (electronic supplementary material, table S5). This result is so clear in our data that it seems surprising that it is not an established finding in the social sciences. This might be due to most social science research focusing on linear relations, and undersampling of individuals who are below the threshold. We look forward to future studies of the desperation threshold in other datasets on risk taking and future discounting as well as other domains of cognition and behaviour.

Our finding that poverty is associated with both risk avoidance and risk taking is important for several reasons. First, as noted, it reconciles two opposing perspectives on poverty and risk taking, which [1] named ‘vulnerability’ and ‘desperation’. In our sample, a larger proportion of individuals living in situations of poverty avoid risk, suggesting that they have ‘too much to lose’. At the same time, a larger proportion declare themselves ready to take risks that are on average detrimental, suggesting they have ‘little to lose’. We also proposed an explanation for why poverty could lead to either vulnerability or desperation: the ‘desperation threshold’, an hypothesis that is analogous to other social sciences theories [32–35,37,38,53]. Our study provides a new source of evidence for the desperation threshold model. Until now, tests of the model have mainly been conducted either (i) in a lab, where poverty (or more precisely, ‘need’) is artificially induced [33,39–44], or (ii) in populations where starvation is a realistic possibility [26–28,47,48]. Our study suggests that a formally equivalent mechanism can apply in the real world to more affluent populations, and that ‘desperate’ risk taking can happen when starvation is unlikely.

The desperation threshold model makes a more precise prediction (P1): individuals should avoid risk just above a ‘desperation threshold’ yet seek risk below it (figure 1B). Most previous real-world studies only searched for an increase in risk taking when poverty increased [27,28,48,54]. In our study, we aimed to simultaneously test the increase and the decrease. Our findings clearly show that both risk taking and risk avoidance were more common among participants with the fewest resources (electronic supplementary material, table S5). Yet, the evidence for a V-shape was less clear: we obtained the predicted V-shape when using our subjective resources measure and a segmented regression model, but not when using our objective resources measure or a polynomial model. In our preregistration, we stated the expectation that we would be less likely to obtain evidence for P1: it requires (i) our resource measure to be precise enough to tell apart individuals just-above the threshold from the ones just-below, and (ii) that the threshold itself does not vary too much between individuals. Still, our Analysis 1 produced conflicting results: we only obtained the predicted V-shape when using subjective resources and a segmented model. Even though we did not anticipate it, we can propose *post-hoc* explanations for this finding. The segmented model might be better suited to test our hypothesis: it fits one relation on only the very bottom part of the resource distribution, while a polynomial regression fits the whole sample at once. Polynomial regressions can also be unreliable for making predictions for extreme values of the independent variable [55], the case we are interested in here.

As for the measure, subjective resources produced more clear-cut results than objective resources in all analyses (figures 2 and 4; electronic supplementary material, table S5, and—on time discounting— electronic supplementary material, table S6). This could mean that the subjective measure is simply a better measure of poverty, and that people are better than researchers at estimating their own situation. In particular, their self-assessment could take into account savings and anticipations of the future, whereas our objective measure did not. This echoes the recurrent finding that subjective socio-economic status is more predictive of health outcomes than objective socio-economic status [56–58]. This result is also reminiscent of a recent finding [59], that in multiple European countries, ‘deprivation ceases to be correlated with income below a certain threshold’ (p. 1). Importantly, the desperation threshold might differ between individuals, as some individuals have higher needs. We tried to capture this in our objective measure, by dividing income by unavoidable costs. Yet, there are likely other ‘needs’ that were not measured. Our subjective measure might better incorporate those needs, since participants estimated for themselves their risk of lacking resources in the near future. Furthermore, our objective resources variable measures flows of resources over a month (income and unavoidable costs), but not stocks (capital). It could thus measure variations in resources, rather than the total amount of resources available, which determines whether an individual can make ends meet. In our sample, 1.6% of the answers have higher unavoidable costs than income over a month. Our objective measure places those answers at the very bottom of the resource distribution. Those points probably reflect an exceptional expense or an unusually low income over one month, which massively influences our objective measure—probably more so than our subjective measure, which should also capture savings and anticipations of the future. Actually, it might be impossible for an extremely poor individual to spend more than she earns, if she has no savings and no options to borrow money. That being said, subjective measures of resources risk are influenced by psychological states, which bring a danger of circularity. It is possible, for instance, that some individuals are panicking because of some unmeasured factor, and therefore report both a higher readiness to take risks and a worse subjective financial situation. In this case, our results still suggest that high financial worries can produce both risk taking and risk avoidance, which is also a new finding.

### (b) Alternative explanations

The desperation threshold model proposes that poverty causes variations in risk taking, but our data only provide evidence for associations. Yet, our finding that populations in poverty are ‘polarized’ in terms of risk taking, with a mixture of risk avoiders and risk takers, enriches the picture of the link between poverty and risk taking.

This result could be produced by different mechanisms. First, causality could be reversed. If risk taking was an entirely stable personality trait, one would expect extreme risk taking or risk aversion to produce a higher chance of poverty. Indeed, some of the most risk-prone individuals would end up very poor as the risks they took have not paid off, while the risk-averse individuals would refuse profitable opportunities, and end up poorer than average. However, risk taking is only weakly-to-moderately stable over time in our data (ICC = 0.48), in line with other findings [60,61]. Moreover, there is evidence that short-term variations in resources can modify risk taking. Using the same data and measures [49], we found evidence that within-person reduction in the objective resources variable was associated with within-person reduction in risk taking. Recently, Akesaka *et al.* [46] also found that individuals most dependent on social security were ready to take more risks the week before welfare checks arrived.

Poverty could also produce our results through a different mechanism. For instance, a lower education or a lower cognitive capacity due to financial stress [62] could lead individuals with fewer resources to not understand the risk questions as well. However, we did not find any evidence of an association between resources and consistency in risk answers (§4d). This class of explanation would also predict that individuals in poverty misunderstand other questions as well and display extreme scores in other domains than risk taking. In our data, the ‘time discounting’ questions were similar in terms of language and allow for comparison. To test for this alternative explanation, we replicated our exploratory analysis using time discounting. Our results (§4d) suggest that among the most deprived participants, steep time discounting was more frequent, but flat time discounting less frequent, whereas the alternative explanation (i.e. more errors among participants with low resources) would predict both to be more frequent.

Our results could also be driven by measurement error: some participants may fill the survey less seriously and report extreme levels of both resources and risk taking, in either direction. But if so, we would find the same phenomenon not only on time discounting but also among the individuals with high objective resources. This was not the case: the top 5% in objective and subjective resources showed a lower variance in risk taking and provided fewer extreme answers (electronic supplementary material, table S5).

### (c) Limitations

The Changing Cost of Living sample was not representative of UK or French populations. There were no participants below the age of 25, and few over 45. Also, the recruitment via online participation platforms produced an oversampling of individuals with low incomes (for details, see [49]). This could have been an advantage to test our hypothesis, which requires a sufficient number of low-income individuals to detect the pattern. To evaluate how generalizable our results are, future research should test our predictions in other populations, ideally from different regions with different levels of standard of living.

Our risk taking measure also has limitations. Hypothetical lottery measures may have suboptimal external validity. They predict behaviours like portfolio choice, occupational choice, smoking and migration [20], but less well than ‘general risk questions’, like ‘Are you generally a risk taking person or do you try to avoid risks?’ [20,63]. This second measure also tends to be more stable over time and has a higher ‘convergent validity’—that is, better generalizes across domains of risk taking [20]. However, the ‘desperation threshold’ only applies to risks related to resources. It can make a clear prediction on hypothetical lotteries (figure 1B), but not on the general risk questions. Moreover, because our goal was to capture risk taking as a response to current material conditions rather than a lasting personality trait, the lower temporal stability is thus not an issue for our research question. The hypothetical gambles thus seemed appropriate for our study, even if imperfect, for example because they were not actually incentivized.

### (d) Implications

Our study has important societal implications, both to explain and to remedy problems associated with poverty. In our data, people in poverty were more likely to (i) avoid risk even when it would, on average, benefit them, and to (ii) take risks even when it will, on average, be detrimental. In both cases, such individuals are further from ‘expected payoff’ decision-making, which is, by definition, optimal if one wants to maximize resources in the long term. In a way, the desperation threshold makes it optimal to make decisions that are long-term sub-optimal from a poverty-reduction perspective.

Concretely, Banerjee [1] points out that both ‘poverty as vulnerability’ and ‘poverty as desperation’ can lock people in poverty: if people in poverty have too much to lose, they refrain from investing; if they have little to lose, they have ‘no obvious reason to want to repay’ (p. 62) a loan, and therefore no one would lend them resources. In both cases, it is harder for them to escape poverty. In previous research in economics, risk aversion has often been deemed as the cause of suboptimal decisions—in particular in agricultural economics, where it was proposed as the cause of field scattering (e.g. [64]) or refusal to adopt new, more profitable, technologies [65].

‘Desperate risk taking’ likely imposes major costs on individuals, communities and society at large. When below the desperation threshold, our model predicts that people will take risks even when they have a negative expected payoff (figure 1B). In our data, the proportion of participants ready to take such ‘bad risks’ was twice as high in the lowest 5% in subjective resources (electronic supplementary material, table S5). In reality, risks that people in poverty have access to are likely to fall into this category: they lack the money to invest in risky but profitable assets, and can only borrow with astronomically high interest rates [66]. Also, a desperate individual needs resources urgently, to fulfill a basic need. One way to obtain resources quickly without investing might be to engage in property crime. It is a particularly risky activity: it implies the fundamental uncertainty of being caught and punished.

In some cases, it is thus plausible that desperate risk taking takes the form of crime. Empirically, risk taking (measured by hypothetical lotteries) has indeed been found to strongly predict property crime [67]. Crime (and in particular property crime) is more frequent in deprived [13] or unequal [68,69] populations, a phenomenon that some attribute to a ‘little to lose’ feeling [12,70], or to ‘a mind-set in which offenders are seeking less to maximize their gains than to deal with a present crisis’ ([71], p. 167).

However, if we equate willingness to take risks and willingness to engage in property crime, our model and our data have a counter-intuitive prediction. It is possible that people in poverty are, on average, more law-abiding (risk taking is on average lower), and yet, most crime occurs there, since people ready to take extreme risks are mostly found among them (figure 2). This could, in turn, create discrimination: people in poverty could be suspected and mistrusted more, even though the majority of them are on the contrary especially unlikely to engage in crime. In other words, the fact that a minority of people in poverty are in a situation where they have to take risks might create a stigma affecting other people also in poverty. This could generate the fact that poorer people are, empirically, trusted less [72], even though they might be less likely to engage in unethical behaviour [73,74].

Finally, the desperation threshold has implications for the welfare system. By helping to meet basic needs under any conditions, social security measures—for instance, unemployment benefits or health insurance—should alleviate the desperation thresholds, and therefore ‘smoothen’ individuals’ utility function. This should reduce both extreme risk aversion (one has less to lose if there is a strong safety net) and extreme risk taking (desperation would become rarer, or non-existent). Empirically, both risk aversion [75] and crime rates [76] tend to be lower in countries that have a stronger welfare state, which may indicate that such smoothing indeed takes place.

## Data Availability

The data are available at OSF [77]. This article was written in R markdown, which makes the analyses and the plots reproducible inside the document. The code, and the Python code used to produce figure 1, can be found at https://github.com/regicid/changing_cost_of_living_desperation. Supplementary material is available online [78].
